# Neurophysiologic Profiling of At-Risk Low and Very Low Birth-Weight Infants Using Magnetic Resonance Imaging

**DOI:** 10.3389/fphys.2021.638868

**Published:** 2021-03-23

**Authors:** Ying Qi, Jingni He

**Affiliations:** ^1^Department of Radiology, Shengjing Hospital of China Medical University, Shenyang, China; ^2^Department of Surgery, Shengjing Hospital of China Medical University, Shenyang, China

**Keywords:** low birth weight (LBW), very low birth weight (VLBW), PC MRI, TRUST MRI, CMRO_2_, CBF, OEF

## Abstract

Low birth-weight (LBW) and very low birth-weight (VLBW) newborns have increased risks of brain injuries, growth failure, motor difficulties, developmental coordination disorders or delay, and adult-onset vascular diseases. However, relatively little is known of the neurobiologic underpinnings. To clarify the pathophysiologic vulnerabilities of such neonates, we applied several advanced techniques for assessing brain physiology, namely T2-relaxation-under-spin-tagging (TRUST) magnetic resonance imaging (MRI) and phase-contrast (PC) MRI. This enabled quantification of oxygen extraction fraction (OEF), global cerebral blood flow (CBF), and cerebral metabolic rate of oxygen (CMRO_2_). A total of 50 neonates (LBW-VLBW, 41; term controls, 9) participated in this study. LBW-VLBW neonates were further stratified as those with (LBW-VLBW-a, 24) and without (LBW-VLBW-n, 17) structural MRI (sMRI) abnormalities. TRUST and PC MRI studies were undertaken to determine OEF, CBF, and CMRO_2_. Ultimately, CMRO_2_ proved significantly lower (*p* = 0.01) in LBW-VLBW (vs term) neonates, both LBW-VLBW-a and LBW-VLBW-n subsets showing significantly greater physiologic deficits than term controls (*p* = 0.03 and *p* = 0.04, respectively). CMRO_2_ and CBF in LBW-VLBW-a and LBW-VLBW-n subsets did not differ significantly (*p* > 0.05), although OEF showed a tendency to diverge (*p* = 0.15). However, OEF values in the LBW-VLBW-n subset differed significantly from those of term controls (*p* = 0.02). Compared with brain volume or body weight, these physiologic parameters yield higher area-under-the-curve (AUC) values for distinguishing neonates of the LBW-VLBW-a subset. The latter displayed distinct cerebral metabolic and hemodynamic, whereas changes were marginal in the LBW-VLBW-n subset (i.e., higher OEF and lower CBF and CMRO_2_) by comparison. Physiologic imaging may therefore be useful in identifying LBW-VLBW newborns at high risk of irreversible brain damage.

## Introduction

Preterm newborns of low birth weight (LBW, ≤2,500 g) or very low birth weight (VLBW, ≤1,500 g) present a substantial public health problem. In 2015, the worldwide prevalence was 14.6%, with 91% confined to low- and middle-income countries [primarily Southern Asia (24%) and sub-Saharan Africa (48%)]. More than 80% of perinatal deaths occur in LBW neonates (Blencowe et al., 2019).

Although most LBW and VLBW preterm infants show catch-up gains in height and weight, they are still at increased risk of brain injuries (Martinussen et al., 2005), growth failure (Tchamo et al., 2016), motor difficulties, developmental coordination disorders or delay (Burns et al., 2009), adult-onset vascular diseases (Blencowe et al., 2013; Christian et al., 2013), and type 2 diabetes (Barker, 2003). It also appears that catch-up or postnatal accelerated growth in LBW and VLBW neonates may adversely affect cognitive function (Estourgie-van Burk et al., 2009). Consequently, better understanding of neonatal brain functions, particularly cerebral oxygen metabolism and hemodynamics, may provide important pathophysiologic insights in this setting.

Because neonatal brain sizes are small and highly vulnerable in terms of motion and scanning times, physiologic and functional brain imaging is especially challenging. ^133^Xenon clearance (Colditz et al., 1988), computed tomography (CT; Dani et al., 2012), positron emission tomography (PET; Wright et al., 2016), and near-infrared spectroscopy (NIRS; Kusaka et al., 2014; Hashem et al., 2020) are common methods used to measure metabolism and cerebral blood flow (CBF) in adults. Unfortunately, the above involve radiation exposure, present difficulties when imaging deep brain tissues, or require exogenous tracers. As a result, their use in neonatal brain imaging is limited.

Recent advances in magnetic resonance imaging (MRI) technologies have allowed noninvasive and quantitative measurements of critical neurophysiologic parameters, without need of contrast agents. The oxygen extraction fraction (OEF) of the brain is assessable using T2-relaxation-under-spin-tagging (TRUST) MRI technique (Liu et al., 2014), and global CBF may be measured using quantitative flow phase-contrast (PC) MRI. OEF and CBF may then be combined to determine the global cerebral metabolic rate of oxygen (CMRO_2_; Liu et al., 2014). Importantly, these physiologic measures are fully obtained in <5 min, making them particularly suitable for neonatal testing (Qi et al., 2018; Shetty et al., 2019).

Arterial spin labeling (ASL) is another valuable means of perfusion MRI for this purpose. However, its signal-to-noise ratio (SNR) tends to be lower, the scan times lengthier, and there is a potential for confounding factors (i.e., bolus arrival time). In an earlier effort, infants with hypoxic ischemic encephalopathy (HIE) showed lower CBF and CMRO_2_ using T2 prepared tissue relaxation inversion recovery (T2-TRIR) pulse sequences and ASL (De Vis et al., 2014). Newborns with severe HIE have also registered lower CBF and extracted less oxygen than those with moderate HIE using ASL and NIRS, a strong correlation found between CMRO_2_ and CBF in asphyxiated newborns with severe HIE (*r* = 0.88) (Wintermark et al., 2014). Our previous look at newborns with white matter damage indicated lower oxygen consumption and CBF using PC MRI and TRUST MRI (Qi et al., 2018). Tortora has similarly reported on CBF (via ASL) in preterm neonates (birth weights, 1,317–2,250 g; postmenstrual ages, 36–39 weeks) with low-grade germinal matrix-intraventricular hemorrhage (GMH-IVH), finding significantly lower rates than in preterm neonates (birth weights, 1,160–1,680 g; postmenstrual ages, 38–41 weeks) without MRI abnormalities (Tortora et al., 2020). There have been few perinatal studies of cerebral oxygen metabolism and hemodynamics in LBW and VLBW preterm infants, especially at postmenstrual ages before term and taking structural abnormalities into account.

In the present study, we used TRUST MRI and PC MRI to determine OEF, CBF, and CMRO_2_ in a mixed group of LBW and VLBW newborns, comparing outcomes with those of term neonates.

## Materials and Methods

### Study Population

Our investigational protocol received approval from the Ethics Committee at Shengjing Hospital of China Medical University. Between June 2016 and December 2018, a total of 50 newborns accrued for study. The structural MRI pulse sequences performed included axial and sagittal T1-weighted imaging (TIWI), axial T2-weighted imaging (T2WI), and diffusion-weighted imaging (DWI). Congenital malformations, severe infections, or unusable MRI studies were grounds for exclusion. Subjects qualified as LBW-VLBW (*n* = 41) at birth weights >1,000 g but <2,500 g. Term neonates serving as controls (*n* = 9) had birth weights ≥2,500 g, were ≥37 weeks at birth, and showed no sMRI abnormalities. Population characteristics and results of blood gas analysis were listed in Table 1. All LBW-VLBW infants were preterm, grouped as those with (LBW-VLBW-a) or without (LBW-VLBW-n) structural MRI abnormalities. Doing so approximates the needs of pediatricians. During perinatal periods, there are often no neurobehavioral differences in preterm LBW and full-term babies (Silva et al., 2018). MRI is frequently used to determine neonatal structural brain damage, assessing type and extent of injury, gray and white matter volumes, and prognostic status. These studies aid in diagnostic accuracy, helping pediatricians formulate helpful and safe treatments and develop long-term follow-up plans.

**TABLE 1:** Clinical characteristics of low birth-weight/very low birth-weight (LBW-VLBW) and term neonates.

		**LBW-VLBW (*n* = 41)**	**Term (*n* = 9)**	**Z/χ^2^**	***p* value**
Characteristics	Males	27 (65.9)	7 (77.8)	6.5	0.011*
	Birth weight, g	1709.2 ± 404.5	3461.1 ± 755.7	−9.9	<0.001*
	Birth age, weeks	32.1 ± 2.4	39.0 ± 1.0	−13.7	<0.001*
	Scan age, weeks	35.3 ± 2.3	40.8 ± 2.1	−6.5	<0.001*
Blood gas analysis	Total tested	36 (87.8)	8 (88.9)		
	pH	7.4 ± 0.06	7.4 ± 0.06	1.60	0.11
	PCO_2_, mmHg	43.2 ± 7.3	40.8 ± 6.2	1.0	0.34
	PaO_2_, mmHg	87.1 ± 6.8	90.4 ± 2.0	−1.6	0.12
	SaO_2_, %	94.8 ± 6.3	96.8 ± 1.5	−0.1	0.92
	Glucose, mmol/L	4.6 ± 1.2	4.6 ± 0.7	0.1	0.91

Data expressed as n (% total) or mean ± standard deviation.

*P < 0.05.

*PCO_2_, partial pressure of carbon dioxide; PaO_2_, partial pressure of oxygen; SaO_2_, oxygen saturation.*

Conventional MR images (including T1WI, T2WI and DWI) obtained from all infants (LBW-VLBW and term) were viewed as Picture Archiving and Communication Systems (PACS) files by two neuroradiologists (YQ and XYS) with 10 years of experience, each blinded to group data. They independently scored MRI abnormalities for each infant, using a method similar to one already described (Woodward et al., 2006) that focuses on six components: gray and white matter signal intensity on T1WI and T2WI, volume of periventricular white matter, presence of blood product deposition or cysts, ventricular dilation, abnormalities on DWI, and corpus callosum thickness. Each aspect was scored (1–4) accordingly (normal, 1; mild, 2; moderate, 3; severe, 4). MRI scores were the sum of the six categorical scores, averaged. Normal brains would thus receive average scores of 6, higher figures indicating abnormalities of commensurate severity.

### General MRI Protocol

A 3T MRI system equipped with a phased-array head coil (Intera Achieva; Philips Healthcare, Best, Netherlands) was used for all MR scans. Prior to imaging procedures, a pediatrician sedated infants through nasal feedings of chloral hydrate (50 mg/kg). This is standard clinical practice at our institution. All newborns were well fed, visually monitored, and hearing protected during scans.

Axial T1-weighted spin-echo images were acquired at the following settings: repetition time/echo time (TR/TE), 200 ms/2.3 ms; section thickness, 5 mm; field of view (FOV), 180 mm × 150 mm × 89 mm; matrix size, 224 × 162; and scan time, 34.4 s. For sagittal T1-weighted spin-echo images, the following settings were used: TR/TE, 250 ms/2.3 ms; section thickness, 5 mm; FOV, 230 mm × 230 mm × 107 mm; matrix size, 256 × 250; and scan time, 33.0 s. Axial T2WI used the following settings: TR/TE, 5,000 ms/80 ms; section thickness, 5 mm; FOV, 180 mm × 150 mm × 90 mm; matrix size, 112 × 112; and scan time, 40.9 s. Axial echo-planar imaging (EPI) DWI was performed as follows: TR/TE, 3,500 ms/63 ms; section thickness, 5 mm; FOV, 180 mm × 180 mm × 89 mm; b values, 0 and 1,000 s/mm^2^; and scan time, 30 s.

### Oxygen Extraction Fraction Determination

**FIGURE 1:**
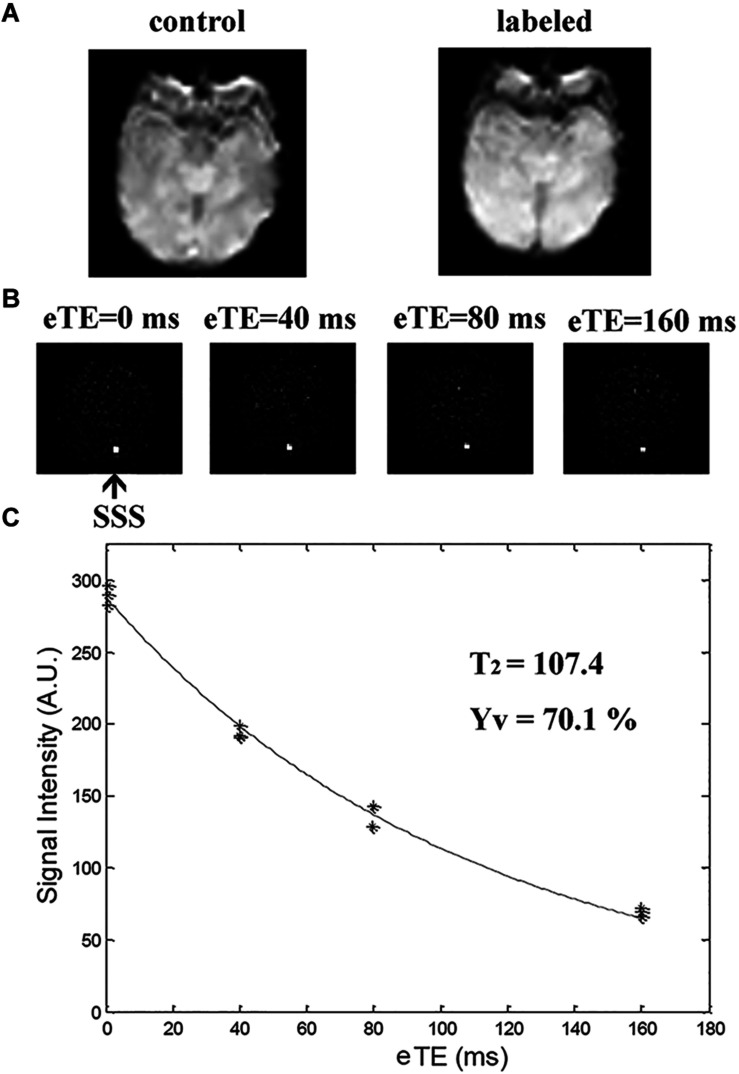
T2-relaxation-under-spin-tagging (TRUST) MRI for quantifying venous oxygenation (Yv) in superior sagittal sinus (SSS). **(A)** Raw images of control and labeled scans; **(B)** Various images (i.e., control-labeled) as function of T2-weighting [effective echo times (eTEs) of 0, 40, 80, and 160 ms; black arrow at SSS); and **(C)** Blood signals in SSS of TRUST MRI fitted to monoexponential function of eTE to yield blood T_2_, in turn converted to Yv via calibration plot.

Oxygen extraction fraction was calculated as follows:

(1) OEF=(Ya-Yv)/Ya×100%

where Ya and Yv signify arterial and venous oxygenation, respectively. Ya was measured peripherally, via pulse oximeter applied to neonatal toes. Yv was measured by TRUST MRI technique, given that blood T2 has a calibrationable relation with oxygenation level. This sequence utilizes a spin-labeling module to isolate pure venous blood signals, thereafter applying a series of T2-preparation pulses to modulate the MRI signal, the monoexponential fitting of which yields blood T2 (Figure 1). A T2-Yv calibration curve served to convert venous T2 to blood oxygenation, using clinically determined hematocrit values of newborns (Liu et al., 2016). Imaging slices selected for TRUST MRI were based on existing procedures (Liu et al., 2014; Qi et al., 2018), acquired parallel to intercommissural lines at a 10-mm distance from sinus confluence [postmenstrual age (PMA) ≥ 36 weeks] or directly through sinus confluence (PMA < 36 weeks). Other settings were as follows: labeling slab thickness, 80 mm; effective TEs (eTEs), four (0, 40, 80, and 160 ms); TR, 3000 ms; inversion time (TI), 1022 ms; FOV, 160 mm × 160 mm × 5 mm; matrix size, 64 × 61; SENSE factor, 3; voxel size, 2.5 mm × 2.5 mm × 5 mm; τCPMG, 10 ms; eTE executions, three pairs; and total scan time, 72 s. Processing of TRUST MRI data adhered to a previously established format (Liu et al., 2014; Qi et al., 2018; Shetty et al., 2019). Global Yv values and Ya values together provided OEF estimates.

### Cerebral Blood Flow Determination

For measuring CBF, PC MRI employed bipolar gradients to encode flow velocities of blood supplying major feeding arteries of the brain, specifically left and right internal carotid arteries (LICA and RICA) and left and right vertebral arteries (LVA and RVA), allowing quantitation of total blood flow to the brain. Flow values were then normalized to brain volume, expressing CBF in units of mL/100 g/min. Time-of-flight MR angiograms (TOF-MRAs) were first performed to allow visualization of above-referenced feeding arteries. Imaging slabs were positioned at center of epistropheus, with 60-mm saturation slabs placed above to suppress venous signals. Settings of TOF MRA were as follows: TR/TE, 20 ms/3.45 ms; flip angle, 18°; FOV, 90 mm × 90 mm × 20 mm; voxel size, 0.8 mm × 0.8 mm × 2 mm; and scan duration, 23.7 s.

**FIGURE 2:**
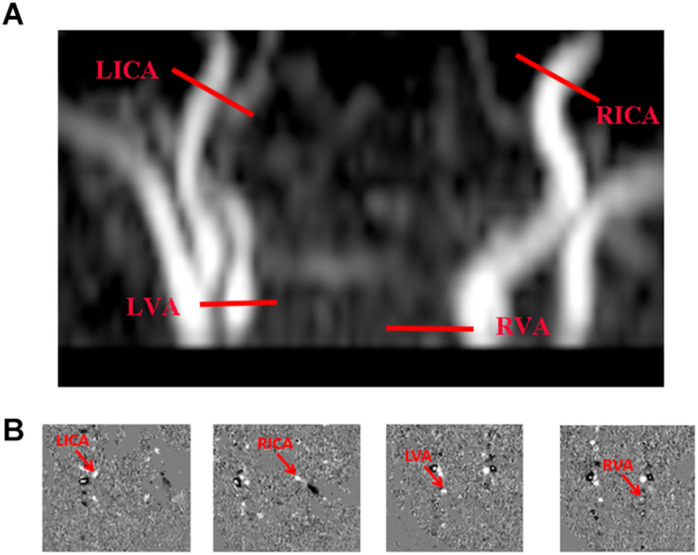
Cerebral blood flow determination via phase-contrast (PC) MRI. **(A)** Slice positions overlaid on angiogram image (each red bar indicating imaging slice of one PC MRI scan), performing four PC MRI scans in each subject to assess internal carotid arteries (left, LICA; right, RICA) and vertebral arteries (left, LVA; right, RVA), respectively; and **(B)** Corresponding PC MRI images (delayed).

Based on MRA images, four PC MRI scans were performed by orienting imaging slices perpendicular to and centered on respective target arteries, as previously described (Liu et al., 2014; Qi et al., 2018). Scans of LICA and RICA took place at level of foramen magnum, whereas the LVA and RVA imaging slices were placed immediately below epistropheus, away from pivot points (Figure 2) and at the following settings: slices, single; voxel size, 0.5 mm × 0.5 mm × 3 mm; FOV, 180 mm × 180 mm × 3 mm; maximum velocity encoding, 20 cm/s (non-gated); and averages, 2. Total scan duration for four arteries was 1.5 min.

Processing of PC MRI data again followed existing procedures (Liu et al., 2014; Shetty et al., 2019). Manual delineations of LICA, RICA, LVA, and RVA were achievable on magnitude images of respective PC scans, gauging levels of vascular flux by applying ROI masks to velocity maps (Figure 2). The sum of fluxes in all vessels ultimately yielded whole-brain blood flow (mL/min). To convert this value to CBF, we determined brain volume through manual tracing of T2WI views, based on presumptive parenchymal density of 1.06 g/mL (Liu et al., 2016). CBF was then calculated as total flux divided by brain weight, expressed in mL/100 g/min.

### CMRO_2_ Calculation

Once OEF and CBF were determined, CMRO_2_ was calculated according to Fick’s principle (Herscovitch et al., 1985; Burgess et al., 2018):

(2) $${\text{CMRO}}_{2}=\text{CBF}\times \text{OEF}\times \text{Ya}\times \text{Ca},$$

where Ca is the oxygen-carrying capacity of blood, presumptively 897 μmol O_2_/100 mL at a hematocrit of 44% (Liu et al., 2013), and CMRO_2_ is expressed as μmol O_2_/100 g/min. Neonatal Ca values were subject-specific, based on individual hematocrit levels.

### Statistical Analysis

Linear regression analysis was undertaken to examine reliance of physiologic parameters (dependent variables) on scan age, sex, and diagnostic group (independent variables). In regression analyses, paired groups were encoded as follows: LBW-VLBW (0) vs term (1) neonates; LBW-VLBW-a (1) vs LBW-VLBW-n (0) subsets; LBW-VLBW-a neonates (1) vs term controls (0); and LBW-VLBW-n neonates (0) vs term controls (1). LBW-VLBW newborns underwent MRI scans once their vital signs had stabilized, and ventilators were no longer required (3 weeks old on average). Term infants had good vital signs and were amenable to scanning at any time (1 week old on average). There were distinct chronologic differences in scan ages of these pairings. Due to remarkable disparities (Table 1), it was necessary to correct birth weight, CMRO_2_, CBF, OEF, and brain volume determinations of LBW-VLBW and term neonates for scan age and sex prior to comparisons. For instance, corrected OEF was calculated as follows:

(3) $$\mathrm{Corrected}\,\mathrm{OEF}=\mathrm{OEF}-b_{1}\times \left(\mathrm{age}-\bar{\text{age}}\right)-b_{2}\times \left(\mathrm{sex}-\bar{\text{sex}}\right)$$

where coefficients b_1_ and b_2_ were based on results of an age- and sex-adjusted regression model similarly reported (Peng et al., 2014). Corrected birth weights, other physiologic parameters, and brain volumes of LBW-VLBW and term neonates were compared using independent-samples *t-*test, separately comparing LBW-VLBW (*n* = 41) and term neonates (*n* = 9), LBW-VLBW-a (*n* = 24) and LBW-VLBW-n (*n* = 17) subsets, LBW-VLBW-a neonates (*n* = 24) and term controls (*n* = 24), and LBW-VLBW-n neonates (*n* = 17) and term controls (*n* = 24). We also analyzed birth weight, brain volume, and cerebral physiologic parameters independently, generating receiver operating characteristic (ROC) curves to distinguish the LBW-VLBW-a subset from all other neonates and from the LBW-VLBW group overall. Inter-rater variations in MRI scores determined by YQ and XYS were assessed via intraclass correlation coefficient (ICC), rated as follows: 1.0–0.81, excellent; 0.80–0.61, very good; 0.60–0.41, good; 0.40–0.21, reasonable; and 0.20–0.00, poor. All computations were driven by standard software (SPSS v21; IBM Corp, Armonk, NY, United States), setting significance at *p* < 0.05.

## Results

### Characteristics of Study Population

In LBW-VLBW and term neonates, measured parameters were as follows: OEF, 31.3 ± 10.5% vs 30.8 ± 6.2% (*p* = 0.89); CMRO_2_, 29.9 ± 11.4 μmol/100 g/min vs 48.9 ± 12.4 μmol/100 g/min (*p* < 0.001); CBF, 13.6 ± 5.0 mL/100 g/min vs 19.5 ± 3.8 mL/100 g/min (*p* = 0.002); and brain volume, 265.2 ± 74.5 mL vs 354.1 ± 52.0 mL (*p* = 0.001). Only OEF proved similar, the other parameters differing significantly by group.

**FIGURE 3:**
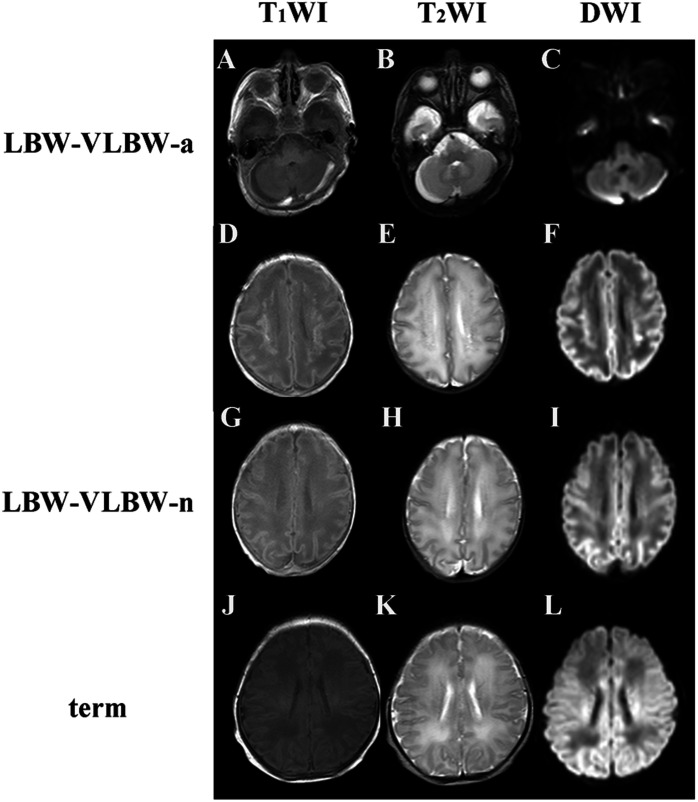
T1-, T2-, and diffusion-weighted imaging (T1WI, T2WI, and DWI) studies of low birth-weight/very low birth-weight (LBW–VLBW) and term neonates: **(A–C)** 35^+1^ week-old male newborn (birth weight, 2,000 g) with subarachnoid hemorrhages of posterior cranial fossa (LBW–VLBW-a subset); **(D–F)** 35^+1^ week-old female newborn (birth weight, 2,200 g) with white matter damage and periventricular leukomalacia (LBW–VLBW-a subset), showing multiple increased signal intensities on T1WI and DWI, decreased signal intensity on T2WI, and cystic lesions of centrum semiovale bilaterally; **(G–I)** 34^+5^ week-old male newborn (birth weight, 1,625 g) with no MRI abnormalities (LBW–VLBW-n subset); and **(J–L)** maps indicating 42^+3^ week-old male term newborn (birth weight, 3,400 g) with no MRI abnormalities.

Population characteristics and diagnostic outcomes (i.e., MRI findings, blood gas results, and physiologic parameters) of the LBW-VLBW group (*n* = 41), including both LBW-VLBW-a and LBW-VLBW-n subsets, are detailed in Table 2 and Figures 3A–I. Term controls included nine neonates, each undergoing clinically indicated MRI based on certain clinical signs/symptoms, umbilical cord blood gas analysis, or other routine blood testing at birth. However, no MRI abnormalities were observed, ostensibly excluding any nervous system diseases (Figures 3J–L). The inter-rater ICC value of MRI scoring was 0.9, indicating excellent reliability. There were six infants lacking blood gas analysis within 24 h after MRI scans. All values were similar for the LBW-VLBW and term groups and both LBW-VLBW subsets (LBW-VLBW-a and LBW-VLBW-n) (*p* > 0.05). Fifteen LBW-VLBW newborns had required mechanical ventilation.

**TABLE 2:** Clinical characteristics and diagnostic outcomes of LBW-VLBW-a and LBW-VLBW-n neonatal subsets.

		**LBW-VLBW-a**	**LBW-VLBW-n**	**Z/χ^2^**	***p*-Value**
	**(*n* = 24)**	**(*n* = 17)**			
**Clinical characteristics**	Males	13 (54.2)	14 (82.4)	−4.1	0.04*
	VLBW	16 (66.7)	6 (35.3)	0.2	0.64
	Birth weight, g	1788.2 ± 437.9	1597.8 ± 332.9	1.6	0.12
	Birth age, weeks	32.4 ± 2.4	31.8 ± 2.5	0.7	0.49
	Scan age, weeks	35.6 ± 2.3	35.0 ± 2.4	0.8	0.42
	Premature rupture of membranes	6 (25.0)	3 (17.6)		
	Twins	2 (8.3)	1 (5.9)		
	Umbilical cord around neck	1 (4.2)	1 (5.9)		
	Placenta previa	1 (4.2)	0		
	Fetal growth restriction	0	1 (5.9)		
	Disappearance of umbilical blood flow	0	1 (5.9)		
	Reverse umbilical blood flow	0	1 (5.9)		
**MRI findings**	White matter damage	20 (83.3)	0		
	Subarachnoid hemorrhage	2 (8.3)	0		
	Intraventricular hemorrhage (IVH)	1 (4.2)	0		
	Cerebral infarction	1 (4.2)	0		
**Blood gas analysis**	Total tested	21 (87.5)	15 (88.2)		
	pH	7.4 ± 0.06	7.4 ± 0.06	0.4	0.67
	PCO_2_, mmHg	41.9 ± 7.3	45.0 ± 7.2	−1.2	0.22
	PaO_2_, mmHg	87.9 ± 7.0	86.0 ± 6.6	0.8	0.42
	SaO_2_, %	95.0 ± 7.4	94.6 ± 4.7	1.0	0.92
	Glucose, mmol/L	4.5 ± 1.3	4.6 ± 1.1	−1.0	0.30
**Physiologic Parameters**	OEF, %	29.3 ± 11.9	34.2 ± 7.4	−1.5	0.14
	CMRO_2_, μmol/100 g/min	28.2 ± 12.8	32.2 ± 9.1	−1.1	0.28
	CBF, mL/100 g/min	13.1 ± 5.8	14.4 ± 3.5	−0.8	0.41
	Brain volume, mL	273.8 ± 86.1	252.9 ± 54.4	0.9	0.38

Data expressed as *n* (% total) or mean ± standard deviation.

*P < 0.05.

*OEF, oxygen extraction fraction; CMRO_2_, cerebral metabolic rate of oxygen; CBF, cerebral blood flow; LBW-VLBW, low birth-weight/very low birth-weight; LBW-VLBW-a, LBW-VLBW newborns with abnormal structural MRIs; LBW-VLBW-n, LBW-VLBW newborns with normal structural MRIs; PCO_2_, partial pressure of carbon dioxide; PaO_2_, partial pressure of oxygen; SaO_2_, oxygen saturation.*

### Cerebral Metabolism and Hemodynamics According to Group

We first compared physiologic measures of LBW-VLBW and term neonates (Table 3). Although CMRO_2_ was significantly lower (*p* = 0.01) in LBW-VLBW (vs term) newborns, CBF (*p* = 0.06) and OEF (*p* = 0.67) did not differ significantly. Furthermore, CMRO_2_ (*p* = 0.48) and CBF (*p* = 0.63) failed to show scan age-related increases during this period of early development; and despite significant differences in birth weight, LBW-VLBW and term neonates did not differ significantly in brain volume.

**TABLE 3:** Regression analysis of LBW-VLBW (LBW-VLBW-a + LBW-VLBW-n) and term neonates.

		***p*-Value**			**Coefficients (b1,_2_)**			**95% CI of Coefficients**		
		**Scan age**	**Sex**	**Group**	**Scan age**	**Sex**	**Group**	**Scan age**	**Sex**	**Group**
**LBW-VLBW and term neonates**	Birth weight	0.93	0.31	<0.001*	2.8	159.0	−1755.6	−61.4 to 67.0	−150.8 to 468.8	−2265.8 to 1245.4
	OEF	0.67	0.59	0.67	0.3	−1.7	2.3	−1.1 to 1.6	−8.2 to 4.7	−8.3 to 12.8
	CMRO_2_	0.48	0.54	0.01*	0.6	−2.3	−15.7	−1.0 to 2.1	−9.8 to 5.2	−28.1 to 3.4
	CBF	0.63	0.46	0.06	0.2	−1.2	−4.9	−0.5 to 0.8	−4.3 to 2.0	−10.0 to 0.2
	Brain volume	<0.001*	0.10	0.65	18.3	−31.7	14.4	10.3 to 26.2	−70.0 to 6.7	−48.8 to 77.6
**LBW-VLBW-a and LBW-VLBW-n subsets**	Birth weight	0.47	0.63	0.25	21.5	71.4	157.2	−37.6 to 80.7	−225.1 to 368.0	−113.5 to 427.9
	OEF	0.76	0.99	0.15	0.2	0.1	−5.1	−1.3 to 1.8	−7.7 to 7.8	−12.2 to 2.0
	CMRO_2_	0.37	0.74	0.30	0.8	−1.4	−4.0	−0.9 to 2.5	−9.9 to 7.1	−11.8 to 3.8
	CBF	0.75	0.66	0.50	0.1	−0.8	−1.2	0.6 to 0.9	−4.6 to 2.9	−4.6 to 2.3
	Brain volume	<0.001*	0.03	0.30	22.6	−45.4	20.0	14.3 to 31.0	−87.3 to 3.6	−18.2 to 58.2
**LBW-VLBW-a subset and term neonates**	Birth weight	0.74	0.73	<0.001*	15.1	73.9	−806.0	−78.4 to 108.7	−352.1 to 499.9	−1147.4 to 464.5
	OEF	0.64	0.77	0.55	−4.3	1.2	−2.0	−2.3 to 1.4	−7.3 to 9.7	−8.8 to 4.8
	CMRO_2_	0.60	0.99	0.03*	0.6	−0.09	−8.8	−1.6 to 2.8	−10.1 to 10.	−16.9 to 0.8
	CBF	0.77	0.84	0.10	0.1	−0.4	−2.8	−0.8 to 1.1	−4.7 to 3.8	−6.2 to 0.6
	Brain volume	0.004*	0.21	0.62	18.0	−34.2	10.7	6.1 to 29.9	−88.2 to 19.9	−32.6 to 54.0
**LBW-VLBW-n subset and term neonates**	Birth weight	0.34	0.34	<0.001*	−45.4	257.2	−2115.3	−142.4 to 51.5	−285.6 to 800.1	−2828.4 to 1402.3
	OEF	0.03*	0.23	0.02*	1.4	−4.0	11.1	0.2 to 2.6	−10.8 to 2.8	2.2 to 20.0
	CMRO_2_	0.74	0.47	0.04*	0.3	−4.0	−15.1	−1.7 to 2.3	−15.1 to 7.2	−29.7 to 0.4
	CBF	0.41	0.39	0.16	0.3	−1.6	−3.6	−0.4 to 1.0	−5.5 to 2.2	−8.7 to 1.5
	Brain volume	0.02*	0.21	0.25	11.1	−31.8	−38.1	2.0 to 20.2	−82.7 to 19.2	−105.0 to 28.8

*p < 0.05.

*CI, confidence interval; OEF, oxygen extraction fraction; CMRO_2_, cerebral metabolic rate of oxygen; CBF, cerebral blood flow; LBW-VLBW, low birth-weight/very low birth-weight; LBW-VLBW-a, LBW-VLBW newborns with abnormal structural MRIs; LBW-VLBW-n, LBW-VLBW newborns with normal structural MRIs.*

Next, we compared the two subsets of LBW-VLBW neonates (LBW-VLBW-a and LBW-VLBW-n) with term newborns, both displaying lower CMRO_2_ rates relative to controls (*p* = 0.03 and *p* = 0.04, respectively). Contrary to expectations, no significant differences in CMRO_2_ (*p* = 0.30), CBF (*p* = 0.50), or OEF (*p* = 0.15) recordings were evident across subsets upon linear regression analysis. Relative to term neonates, OEF was significantly lower (*p* = 0.02) in the LBW-VLBW-n subset.

**FIGURE 4:**
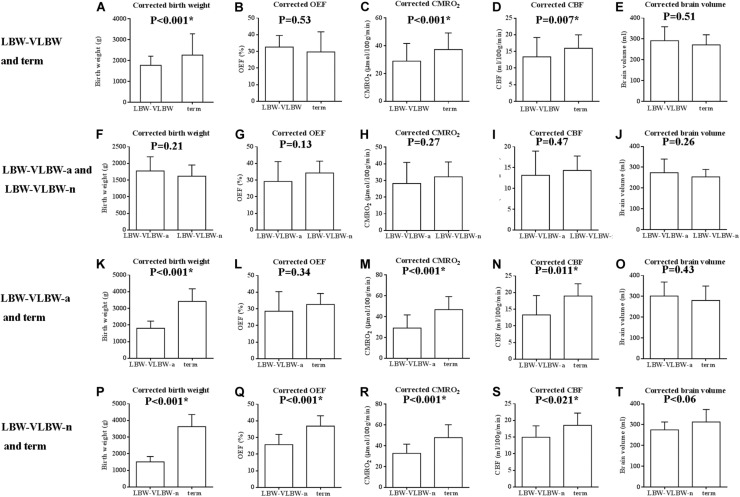
Box plots of corrected birth weight, OEF, cerebral metabolic rate of oxygen (CMRO_2_), cerebral blood flow (CBF), and brain volume, comparing low birth-weight/very low birth-weight (LBW-VLBW) and term newborns, LBW-VLBW-a and LBW-VLBW-n subsets, LBW-VLBW-a subset and term newborns, and LBW-VLBW-n subset and term newborns: **(A,K,P)** Corrected birth weights of LBW-VLBW newborns (+/-MRI abnormalities) exceeded by those of term newborns; **(C,D,M,N,R,S)** Corrected CMRO_2_ and CBF rates differed significantly in LBW-VLBW and term neonates (*p* < 0.001, *p* = 0.007), LBW-VLBW-a subset and term neonates (*p* = 0.001, *p* = 0.011), and LBW-VLBW-n subset and term neonates (*p* < 0.001, *p* = 0.021); **(Q)** Corrected OEF differed significantly in LBW-VLBW-n subset and term neonates (*p* < 0.001); **(F–J)** Corrected values similar (*p* > 0.05) in LBW-VLBW-a and LBW-VLBW-n subsets; and **(B,E,L,O,T)** Other values devoid of significant differences (*p* > 0.05), all data expressed as mean ± standard deviation.

Table 3 lists results of coefficients (b_1_, scan age; b_2_, sex), with 95% confidence interval (CI) of coefficients and respective *p*-values. Once corrected for scan age and sex, group comparisons of birth weight, CMRO_2_, CBF, OEF and brain volume produced similar results (Table 4 and Figure 4). Corrected CMRO_2_ and CBF differed significantly in LBW-VLBW vs term neonates (*p* < 0.001 and *p* = 0.007, respectively), the LBW-VLBW-a subset vs term controls (*p* = 0.001 and *p* = 0.011, respectively), and the LBW-VLBW-n subset vs term controls (*p* < 0.001 and *p* = 0.021, respectively). This suggests that observed differences in physiologic parameters (CMRO_2_ and CBF) of the LBW-VLBW-a subset and term controls stemmed from brain injuries and did not reflect scan age or sex. Corrected OEF values in the LBW-VLBW-n subset did differ significantly (*p* < 0.001) from those of term controls. There were no significant differences in corrected values of LBW-VLBW-a and LBW-VLBW-n subsets (*p* > 0.05).

**TABLE 4:** Corrected parameters for LBW-VLBW neonates/subsets and term controls.

**Comparators**		**Birth weight, g**	**OEF, %**	**CMRO_2_, μmol/100 g/min**	**CBF, mL/100 g/min**	**Brain volume, mL**
**LBW-VLBW**		1708.6 ± 396.9	31.6 ± 10.4	30.5 ± 11.3	13.8 ± 5.0	283.8 ± 56.3
**Term**		3464.2 ± 751.9	29.3 ± 6.0	46.2 ± 12.5	18.7 ± 3.7	269.4 ± 69.9
	**t**	10.0	−0.6	3.7	2.8	−0.7
	***p***	<0.001*	0.53	<0.001*	0.007*	0.51
**LBW-VLBW-a**		1774.4 ± 426.7	29.2 ± 12.0	28.2 ± 12.7	13.2 ± 5.8	273.5 ± 65.5
**LBW-VLBW-n**		1616.7 ± 337.3	34.3 ± 7.1	32.2 ± 8.9	14.3 ± 3.5	253.5 ± 35.9
	**t**	1.3	−1.6	−1.1	−0.7	1.1
	***p***	0.21	0.13	0.27	0.47	0.26
**LBW-VLBW-a**		1804.8 ± 429.2	28.6 ± 11.8	29.1 ± 12.7	13.3 ± 5.8	301.6 ± 67.4
**Term**		3416.7 ± 762.0	32.6 ± 6.6	46.7 ± 12.5	19.0 ± 3.7	280.2 ± 69.5
	**t**	−7.7	−1.0	−3.6	−2.7	0.8
	***p***	<0.001*	0.34	0.001*	0.011*	0.43
**LBW-VLBW-n**		1510.4 ± 320.5	36.9 ± 6.2	32.8 ± 8.8	15.0 ± 3.4	274.8 ± 38.2
**Term**		3625.9 ± 729.6	25.7 ± 6.1	47.8 ± 12.4	18.5 ± 3.7	312.9 ± 59.8
	**t**	−10.3	4.4	−3.6	−2.5	−2.0
	***p***	<0.001*	<0.001*	<0.001*	0.021*	0.06

Data expressed as mean ± standard deviation.

*p < 0.05.

*LBW-VLBW, low birth-weight/very low birth-weight; LBW-VLBW-a, LBW-VLBW newborns with abnormal structural MRIs; LBW-VLBW-n, LBW-VLBW newborns with normal structural MRIs.*

### ROC Analysis for Distinguishing LBW-VLBW Neonates With Structural MRI Abnormalities

**FIGURE 5:**
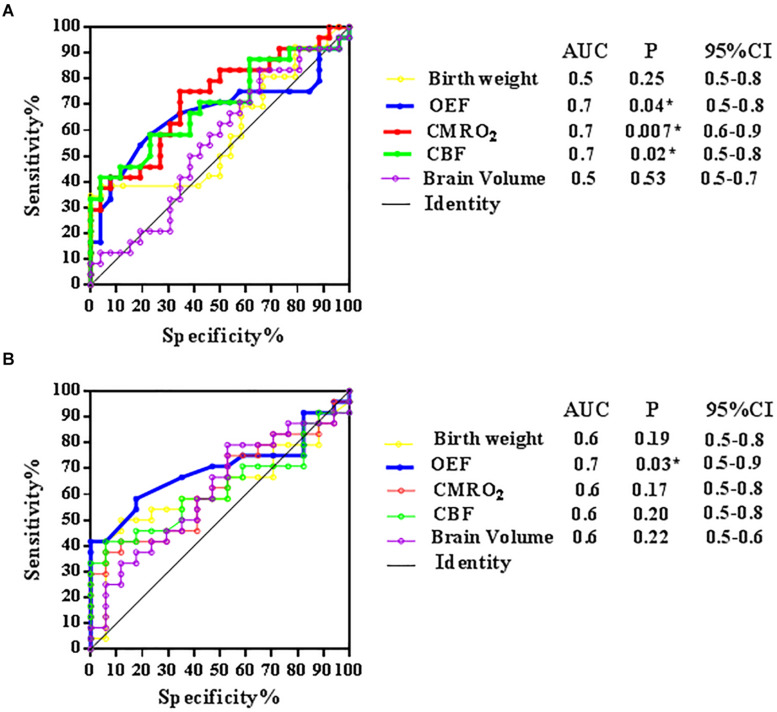
Receiver operating characteristic (ROC) curves for five models [birth weight, oxygen extraction fraction (OEF), cerebral metabolic rate of oxygen (CMRO_2_), cerebral blood flow (CBF), and brain volume] in identifying LBW-VLBW newborns with abnormal structural MRIs (LBW-VLBW-a subset). **(A)** Fair utility of OEF, CMRO_2_, and CBF in distinguishing members of LBW-VLBW-a subset from LBW-VLBW and term newborns and **(B)** Fair utility of OEF only in distinguishing members of LBW-VLBW-a subset from LBW-VLBW neonates. Higher area-under-the-curve (AUC) values for physiologic parameters, compared with morphologic indices of brain weight and volume.

Receiver operating characteristic curves for birth weight [area under the curve (AUC) = 0.6, 95% confidence interval (CI): 0.4–0.8; *p* = 0.25], OEF (AUC = 0.7, 95% CI: 0.5–0.8; *p* = 0.04), CMRO_2_ (AUC = 0.7, 95% CI: 0.6–0.9; *p* = 0.007), CBF (AUC = 0.7, 95% CI: 0.5–0.8; *p* = 0.02), and brain volume (AUC = 0.5, 95% CI: 0.5–0.7; *p* = 0.53) in distinguishing the LBW-VLBW-a subset from term and LBW-VLBW neonates are shown in Figure 5A. OEF, CBF and CMRO_2_ demonstrated fair diagnostic performance in this regard.

Receiver operating characteristic curves for birth weight (AUC = 0.6, 95% CI: 0.5–0.6; *p* = 0.19), OEF (AUC = 0.7, 95% CI: 0.5–0.9; *p* = 0.03), CMRO_2_ (AUC = 0.6, 95% CI: 0.5–0.8; *p* = 0.17), CBF (AUC = 0.6, 95% CI: 0.5–0.8; *p* = 0.20), and brain volume (AUC = 0.6, 95% CI: 0.5–0.6; *p* = 0.22) in distinguishing LBW-VLBW-a neonates from LBW-VLBW neonates are shown in Figure 5B. Interesting, only OEF displayed fair diagnostic performance. MRI physiologic parameters also appeared to outperform brain volume or body weight.

## Discussion

To our knowledge, the present study is the first to evaluate oxygen consumption and CBF in LBW and VLBW preterm neonates using TRUST and PC MRI. Our data suggest that even in the absence of structural abnormalities, LBW and VLBW preterm newborns generally have lower metabolism and CBF, showing pronounced deficits in these physiologic parameters. Compared with conventional measures (i.e., birth weight or brain volume), OEF provided greater power to distinguish LBW-VLBW preterm neonates with *structural* MRI abnormalities.

### Physiologic and Clinical Considerations

A major finding of this study was that LBW-VLBW newborns with normal or abnormal *structural* MRI have similar rates of oxygen consumption and CBF. After correcting for scan age, their CMRO_2_ and CBF rates were still lower than those of healthy term neonates. In reviewing our LBW-VLBW infants overall (regardless of MRI status), we found that most had experienced dyspnea or hypoxemia. Given the abundance of mitochondria in cerebral capillaries, there was likely hypoxic damage to capillary walls, affecting autoregulation of CBF. The passive expansion of vascular channels that results and alters cerebral hemodynamics (Odell et al., 2006).

Premature and LBW newborns also have immature cerebrovascular systems. Once disruptions in oxygen supply and metabolism occur, CMRO_2_ may decline accordingly, increasing the prospect of brain injuries due to poor cerebrovascular function. This perhaps explains the comparatively low CMRO_2_ and CBF rates in both LBW-VLBW-a and LBW-VLBW-n subsets. However, the significantly higher OEF of the LBW-VLBW-n subset (vs term control) is worthy of attention. It may be that once cerebral autoregulation is exhausted by hypoxia, CBF peaks and will change only marginally in response to increased oxygen demand. OEF then progressively increases to maintain constant CMRO_2_ levels. Hence, OEF may be a more comprehensive indicator of tissue-level brain impairment than CBF.

We observed a trend towards reduction of OEF in the LBW-VLBW-a subset, which was not consistent with previous clinical observations. Relative to controls (ductus arteriosus without cerebral pathology: OEF, 25 ± 2%), VLBW preterm neonates with post-hemorrhagic ventricular dilatation (PHVD) have shown significant OEF elevation (36 ± 3%) (McLachlan et al., 2017). Differences in scan age and patient sampling may account for this disparity.

In light of our findings, it seems that normal morphology on brain MRI does not ensure normal metabolic and hemodynamic cerebral physiology in LBW-VLBW preterm newborns. Our data offer support for Laptook’s discovery that nearly 30% of extremely LBW infants with normal head ultrasound studies experienced adverse neurodevelopmental outcomes, including cerebral palsy (Laptook et al., 2005). Furthermore, there are other physiologic variables, namely blood gas analytes (pH, PCO_2_, PaO_2_), glucose concentration, required pressers, or mechanical ventilation, that may impact cerebrovascular hemodynamics at time of MRI. We limited scanning of neonates to those no longer requiring ventilation, whose vital signs were stable. Thus, our blood gas data were similar across groups. Overall, this endeavor was preliminary in nature, forming a basis for continued research on this complex issue.

### Technical Considerations

Phase-contrast MRI and oxygenation MRI have been previously applied to study OEF, CMRO_2_, and CBF in healthy neonates and those with HIE (Liu et al., 2014; Shetty et al., 2019), as summarized in Table 5. The physiologic parameters we measured in term newborns with no *structural* MRI defects were generally aligned with those reported in healthy infants by others (Liu et al., 2014), although our CMRO_2_ and CBF appeared slightly higher (48.9 ± 12.4 mL/100 g/min vs 38.3 ± 17.7 mL/100 g/min; 19.5 ± 3.8 mL/100 g/min vs 13.4 ± 4.2 mL/100 g/min). Differences in scan age and birth weight may have skewed the results to some extent. Compared with our term controls, the LBW-VLBW-a subset displayed lower OEF, CMRO_2_, and CBF determinations, comparable to results reported by Shetty in neonates with HIE (Shetty et al., 2019). The similar OEF, CMRO_2_, and CBF outcomes reached in our LBW-VLBW-n subset with normal *structural* MRIs were also in agreement with values achieved through previous NIRS, ASL, and TRUST studies focused on newborns with hypotensive, PHVD, or HIE conditions (Table 4; Munro et al., 2004; De Vis et al., 2014; McLachlan et al., 2017). Consequently, the accuracy and reliability of TRUST and PC MRI in neonatal assessments of this sort is validated.

**TABLE 5:** Present physiologic measures vs previously published data.

**Study**	**Method**	**Number of subjects and condition**	**Birth weight, g**	**Scan age, weeks**	**OEF, %**	**CMRO_2_, μmol/100g/min**	**CBF, mL/100g/min**
Liu et al., 2014	PC and TRUST	10 healthy	–	37.4 ± 2.6	33.3 ± 2.7	38.3 ± 17.7	13.4 ± 4.2
Shetty et al., 2019	PC and TRUST	5 HIE (hypothermia)	3248 ± 466	39 ± 1.4	24.4 ± 5	34.7 ± 8.3	17.5 ± 6.3
De Vis et al., 2014	ASL and	10 healthy	–	39	49 ± 12	30 ± 6	14 ± 3
	TRUST	9 HIE	–	38	32 ± 12	24 ± 12	12 ± 4
McLachlan et al., 2017	NIRS	9 PHVD	1011 ± 206	26.9 ± 8	36 ± 3	44.6 ± 3.6	14.6 ± 1.0
		13 controls	1035	27	25 ± 2	40.0 ± 6.3	16.5 ± 2.1
Munro et al., 2004	NIRS	12 hypotensive	748 ± 63	25.7 ± 0.4	–	–	14 ± 1
		5 healthy	832 ± 101	27.4 ± 0.9	–	–	19 ± 1
**Present**	PC and TRUST	17 LBW-n	1788.2 ± 437.9	35.0 ± 2.4	34.2 ± 7.	32.2 ± 9.1	14.4 ± 3.5
		24 LBW-a	1597.8 ± 332.9	35.6 ± 2.3	429.3 ± 11.9	28.2 ± 12.8	13.1 ± 5.8
		9 term	3461.1 ± 755.7	40.8 ± 2.1	30.8 ± 6.2	48.9 ± 12.4	19.5 ± 3.8

*Data expressed as mean ± standard deviation.*

*Patent ductus arteriosus identified in13 control neonates, without cerebral pathology.*

*ASL, arterial spin labeling; HIE, hypoxic ischemic encephalopathy; LBW-VLBW, low birth-weight/very low birth-weight; LBW-VLBW-a, LBW-VLBW newborns with abnormal structural MRIs; LBW-VLBW-n, LBW-VLBW newborns with normal structural MRIs; NIRS, near-infrared spectroscopy; PHVD, post-hemorrhagic ventricular dilatation.*

### Relations Between Physiologic Parameters and Scan Age

Unlike previous reports (Liu et al., 2014; Qi et al., 2018), we found that CMRO_2_ and CBF did not increase with scan age. Past investigations may have involved large differences in scan age, birth weight, and cerebral autoregulatory capacities of preterm LBW-VLBW and term newborns.

### AUC Values of Physiologic Parameters in LBW-VLBW Newborns With Structural MRI Abnormalities

Our data indicated that physiologic parameters outperformed morphologic indices of brain volume and birth weight in distinguishing neonates of the LBW-VLBW-a subset, showing higher AUC values. Although T1- and T2-weighted scans readily exposed structural abnormalities in LBW-VLBW neonates, they offered no insights into related pathophysiologic changes and etiologies. Compared with term neonates, the reduced cerebral blood supply and oxygen metabolism observed in perinatal LBW-VLBW infants may be pathophysiologic precursors of morphologic changes, potentially linked to adverse neurodevelopmental outcomes. If OEF significantly declines after decompensation of CBF autoregulation, morphologic MRI abnormalities may then emerge. Our ROC curves confirm that OEF, CMRO_2_, CBF, and especially OEF perform fairly well in distinguishing LBW-VLBW infants with *structural* MRI abnormalities (Figure 5).

In summary, TRUST and PC MRI are useful methods in assessing cerebral oxygen consumption and hemodynamics in LBW-VLBW infants. LBW-VLBW neonates, with or without normal structural MRIs, still have diminished CMRO_2_ and CBF, relative to healthy term neonates of corrected scan age. Abnormal sMRI studies are reflective of diminished OEF, whereas physiologic imaging may help identify LBW-VLBW newborns with sMRI abnormalities and high risk of irreversible brain damage.

### Limitations

It is generally acknowledged that physiologic parameters measured in localized areas of brain injury are more telling than global measurements. Thus, our global measurements may have produced false negatives in group comparisons. On the other hand, such comparisons between preterm LBW-VLBW and full-term healthy infants are apt to be more accurate if scan age is consistent.

Given the worldwide prevalence of LBW-VLBW neonates (∼14.6%), our small population sampling seemed problematic, as did the unrestricted merging of VLBW and LBW neonates, regardless of sex. After appropriate exclusions for congenital malformations, infections, metabolic diseases, and MRI motor artifacts, we were left with only 41 qualifying newborns for the LBW-VLBW group; and the few term neonates recruited as controls required brain scans on a clinical basis [i.e., unusual symptoms/signs, prenatal history, or maternal high-risk factors (pregnancy-induced hypertension, gestational diabetes)]. Some term neonates were also discounted due to abnormal laboratory results or MRI findings. Presently, the potential for neurocognitive deficits in these infants and the relevance of CMRO_2_ or its threshold in relation to such deficits is unknown. CMRO_2_ may be a pivotal determinant of prognosis, helping to formulate reasonable therapeutic strategies.

In future research, we will likely broaden the scope of our study to include neurodevelopmental follow-up of LBW-VLBW neonates, clarifying the impact of CMRO_2_ reductions on neurocognitive deficits. We will also bolster subject recruitment, further stratifying by birth weight (VLBW, LBW) and sex, and foster a multicenter approach to ascertain neurocognitive thresholds for reductions in CMRO_2_. Owing to brain immaturity, especially in VLBW infants, there is exceptional vulnerability to injuries undermining the microstructural integrity and tract connectivity of periventricular white matter (a vascular watershed territory) (Skranes et al., 2007). Knut has reported that VLBW infants exhibit smaller brain volumes and larger lateral ventricles by the age of 20, compared with controls. Diminished gray and white matter volumes and ventricular dilatation in young adults with histories of VLBW may well signal permanent developmental handicaps after perinatal brain injury and impaired cognitive function (Bjuland et al., 2014). We must better address such lesions, adding NIRS to testing protocols for localized CMRO_2_ measurement, with the ultimate goal of cultivating treatment guidelines for the brain injuries that LBW-VLBW infants sustain.

It is unclear at this point whether administering an anesthetic, such as chloral hydrate, has any cerebrovascular hemodynamic ramifications. There is no direct evidence in studies of humans and animals that it increases CBF or lowers glucose metabolism (Grome and McCulloch, 1981; Uematsu et al., 2009). However, investigation of a non-sedated group would be worthwhile, perhaps implementing an immobilization device. Likewise, we used a pulse oximeter affixed to newborn toes in measuring Ya, as others have done (Liu et al., 2014), thus risking possible differences in pulse pressures of upper and lower limbs. The fact that we excluded newborns with congenital malformations (i.e., congenital heart disease) made this scenario less plausible. Going forward, we will use a pulse oximeter on the right hand (pre-ductus measurement) to more accurately reflect SaO_2_ at brain level. Finally, the image quality in Figures 1, 2 was substandard to the rapidity of positioning and scanning. Refinement may yield more positive and interesting findings.

## Conclusion

In the present study, we have demonstrated that structural damage in LBW-VLBW-a neonates is associated with metabolic and hemodynamic deficits of the brain. LBW-VLBW-n neonates with lower CBF and CMRO_2_, and higher OEF tended to show minimal structural abnormalities. Physiologic imaging may be a useful means of identifying those LBW-VLBW newborns at high risk of developing irreversible brain damage.

## Data Availability Statement

The raw data supporting the conclusions of this article will be made available by the authors, without undue reservation.

## Ethics Statement

The study protocol had ethical approval from the Ethics Committee of Shengjing Hospital of China Medical University (IRB2015PS28K). Written informed consent from the participants’ legal guardian/next of kin was not required to participate in this study in accordance with the national legislation and the institutional requirements.

## Author Contributions

YQ contributed study concepts and design, and providing post-processing assistance. Both authors participated in analysis of experiment results, drafting and editing of the manuscript, and have read and approved the final manuscript for publication.

## Conflict of Interest

The authors declare that the research was conducted in the absence of any commercial or financial relationships that could be construed as a potential conflict of interest.
